# Efficacy and Safety of Cystatin C-Guided Renal Dose Adjustment of Cefepime Treatment in Hospitalized Patients with Pneumonia

**DOI:** 10.3390/jcm9092803

**Published:** 2020-08-30

**Authors:** Min-Chul Kim, Seon Ok Kim, Su-Hyun Kim, Jung-ho Shin, Seong-Ho Choi, Jin-Won Chung, Jin Ho Hwang

**Affiliations:** 1 Division of Infectious diseases, Department of Internal Medicine, Chung-Ang University Hospital, Seoul 06973, Korea; kimminchulmd@caumc.or.kr (M.-C.K.); tobeservant@cau.ac.kr (S.-H.C.); drjwchung@cau.ac.kr (J.-W.C.); 2 Biomedical Research Institute, Chung-Ang University College of Medicine, Seoul 06974, Korea; 3 Departments of Clinical Epidemiology and Biostatistics, Asan Medical Center, University of Ulsan College of Medicine, Seoul 05505, Korea; chddk0707@naver.com; 4 Division of Nephrology, Department of Internal Medicine, Chung-Ang University Hospital, Seoul 06973, Korea; sh76so@gmail.com (S.-H.K.); shin1982@caumc.or.kr (J.-h.S.)

**Keywords:** cystatin C, cefepime, mortality, acute kidney injury, cefepime-induced encephalopathy

## Abstract

Cystatin C (CysC) may estimate renal function more accurately than serum creatinine (SCr). The clinical impact of renal dose adjustment of cefepime according to CysC rather than SCr has remained uncertain. We investigated the efficacy and safety of CysC-guided cefepime dosing compared with SCr-guided dosing in hospitalized patients with pneumonia. All adults hospitalized with pneumonia between July 2016 and December 2018 who used cefepime for at least 3 days were enrolled. Mortality, acute kidney injury (AKI), cefepime-induced encephalopathy (CIE), and *Clostridium difficile* infection were compared between the CysC-guided and SCr-guided groups. One hundred and ninety patients were divided into two groups: 129 and 61 received cefepime based on CysC and SCr, respectively. In-hospital mortality did not significantly differ between the groups (12% versus 31%; hazard ratio (HR) 0.74; 95% confidence interval (CI), 0.31–1.77; *p* = 0.50). CysC-guided cefepime dosing decreased the risk of AKI (13% versus 61%; HR 0.18; 95% CI, 0.07–0.44; *p* < 0.001) and CIE (2% versus 11%; HR 0.11; 95% CI, 0.03–0.47; *p* = 0.003) compared with SCr-guided dosing. There was no significant difference in the risk of *Clostridium difficile* infection. CysC-guided dosing of cefepime was associated with decreased risk of the cefepime-associated morbidities including AKI and CIE without increasing mortality among the hospitalized patients with pneumonia.

## 1. Introduction

Cefepime is a broad-spectrum cephalosporin with anti-pseudomonal activity, and it also maintains good potency against gram-positive cocci such as methicillin-susceptible *Staphylococcus aureus* and *Streptococcus pneumoniae* [1]. Cefepime has been recommended for treating community-acquired pneumonia with a risk of *Pseudomonas aeruginosa* infection and hospital-acquired pneumonia in combination with non-beta-lactam antibiotics or alone [2,3]. Cefepime exhibits enhanced stability against AmpC beta-lactamases [4]. A variable portion of extended-spectrum beta-lactamases-producing Enterobacteriaceae have remained susceptible to cefepime in vitro, although failures have been reported in serious infections [5]. In addition, cefepime shows good tissue penetration that reaches adequate intrapulmonary concentration [6]. However, significant adverse drug reactions such as acute kidney injury (AKI) [7,8,9] and cefepime-induced encephalopathy (CIE) [10,11,12,13] have raised safety concern about cefepime use. Therefore, proposing a preventive intervention which could reduce the cefepime-associated morbidities without increasing mortality would have significant implications for clinical practice and antibiotic stewardship program.

Cystatin C (CysC) is considered to be an alternative endogenous marker to serum creatinine (SCr) for estimating glomerular filtration rate (GFR) [14]. While creatinine is produced only in muscle, CysC is produced in all nucleated cells, and the CysC level is not affected by muscle mass and diet [15]. Kidney Disease: Improving Global Outcomes (KDIGO) has recommended the use of the 2012 Chronic Kidney Disease Epidemiology Collaboration (CKD-EPI) creatinine-cystatin C equation to estimate GFR when we have to know more accurate GFR such as confirming a diagnosis of chronic kidney disease (CKD) for the first time and adjusting dosage of possibly toxic drugs that are mainly excreted by kidney [16,17]. However, there have been limited data on the efficacy and safety of the renal dose adjustment of cefepime treatment according to CysC. We therefore investigated the impacts of CysC-guided dosing of cefepime compared to SCr-guided dosing on the mortality and the cefepime-associated morbidities including AKI, CIE, and *Clostridium difficile* infection in treating patients with hospitalized pneumonia using inverse-probability-of-treatment-weighting (IPTW).

## 2. Materials and Methods

### 2.1. Study Patients and Baseline Clinical Characteristics

All adult patients ≥ 18 years of age who were admitted to Chung-Ang University Hospital, an 850-bed tertiary hospital in Seoul, South Korea, from July 2016 to December 2018 for pneumonia treatment were retrospectively analyzed. Patients with pneumonia who were treated with cefepime for at least 3 days or more were enrolled. Subjects who received other antibiotics such as glycopeptides and fluoroquinolones in combination with cefepime were not excluded. In Chung-Ang University Hospital, cefepime dosage is adjusted as recommended [1]. However, whether CysC is to be checked and whether cefepime is to be adjusted according to CysC or SCr in a patient has been decided by clinicians, because there is no definitive guideline for the use of CysC for cefepime dosing. Individuals in whom CysC levels were not checked were excluded because renal dose adjusting method is unclear in these patients. Finally, the enrolled patients were divided into two groups: those who received a renal dose adjustment of cefepime according to the CysC-based eGFR (eGFRcys) and those who received a renal dose adjustment of the drug according to the SCr-based eGFR (eGFRcr). The study protocol was approved by the Chung-Ang University Hospital Institutional Review Board (No. 1908-013-16279).

Baseline clinical characteristics of the patients were checked as follows: age; sex; body mass index (BMI); classification of pneumonia including community-acquired pneumonia, hospital-acquired pneumonia, and ventilator-associated pneumonia; and underlying diseases such as neurologic disease, diabetes, cardiovascular disease, CKD, chronic lung disease, malignancy, and thyroid illness. Laboratory data including white blood cell and platelet counts, blood urea nitrogen, CysC and SCr levels, eGFRcys and eGFRcr, total bilirubin, and C-reactive protein levels were collected at the date of diagnosis of pneumonia. Baseline CysC and SCr levels were defined as the results obtained within 3 days of the enrollment date. Recent CysC level within 2 weeks of the pneumonia diagnosis was considered as the baseline result only if SCr was in a steady state during the period. Additionally, shock, mechanical ventilation, and duration of cefepime and other antibiotics use were reviewed.

### 2.2. Measurement of Cystatin C and Serum Creatinine

CysC was measured using a particle-enhanced turbidimetric immunoassay (Gentian Cystatin C Immunoassay^®^, Gentian AS, Norway) calibrated against the international reference material ERM-DA471/IFCC [18]. SCr was measured using a compensated Jaffe method, standardized against an isotope dilution mass spectrometry method (AU5800^®^, Beckman Coulter, USA). The eGFR was calculated using the CKD-EPI equations according to the measured CysC and SCr as follows [17]: 

2012 CKD-EPI Cystatin C-based eGFR equation:

CysC ≤ 0.8 mg/L = 133 (CysC /0.8)^−0.499^ × 0.996^age^ [0.932 × if female] mL/min/1.73 m^2^

CysC > 0.8 mg/L = 133 (CysC /0.8) ^−1.328^ × 0.996^age^ [0.932 × if female] mL/min/1.73 m^2^

2012 CKD-EPI serum creatinine-based eGFR equation:

Females, SCr ≤ 0.7 mg/dL = 144 × (SCr/0.7)^−0.329^ × 0.993^age^ mL/min/1.73 m^2^

Females, SCr > 0.7 mg/dL = 144 × (SCr/0.7)^−1.209^ × 0.993^age^ mL/min/1.73 m^2^

Males, SCr ≤ 0.9 mg/dL = 141 × (SCr/0.9)^−0.411^ × 0.993^age^ mL/min/1.73 m^2^

Males, SCr > 0.9 mg/dL = 141 × (SCr/0.9)^−1.209^ × 0.993^age^ mL/min/1.73 m^2^

### 2.3. Study Outcomes and Definitions

Between the CysC-guided and the SCr-guided groups, we compared 14-day, 30-day, 60-day, and in-hospital mortality for the evaluation of the efficacy of cefepime treatment. Additionally, we compared the frequency of AKI, CIE, and *Clostridium difficile* infection for the assessment of the safety of cefepime therapy. In-hospital mortality was defined as all-cause death during hospitalization periods. The definition of AKI was compliant with the KDIGO guidelines [19]: stage I, an increase in SCr of 1.5–1.9 times from the baseline or by ≥ 0.3 mg/dL; stage II, an increase in SCr of 2.0–2.9 times from the baseline; stage III, an increase in SCr by more than 3.0 times from the baseline, by ≥ 4.0 mg/dL, or the initiation of renal replacement therapy. Severe AKI was defined as AKI stage II or III. As the previous studies [20,21], AKI was defined as all-cause AKI events that occurred during cefepime treatment or within 72 h of cefepime completion. CIE was diagnosed when the following criteria were met like the previous studies [10,11,12]: (1) neurologic symptoms consistent with encephalopathy including altered mental status, seizure, and non-convulsive status epilepticus that developed during cefepime administration or within 72 h of cessation of the drug; (2) exclusion of alternative causes for the neurological symptoms; and (3) neurological improvement after cefepime discontinuation.

### 2.4. Statistical Analysis

Categorical data were compared using the χ2 or Fisher’s exact test, and continuous variables were analyzed by the *t* test or Mann–Whitney *U* test as appropriate. To evaluate the efficacy and safety of CysC-guided renal dose adjustment of cefepime, we performed rigorous adjustment for significant differences in baseline characteristics of the patients with the weighted Cox proportional-hazards regression models using the IPTW method. Weights for patients receiving SCr-guided dosing were the inverse of (1 – propensity score), and weights for patients receiving CysC-guided dosing were the inverse of the propensity score. The propensity scores were estimated by multiple logistic-regression analysis. All baseline clinical characteristics (Table 1), with the exception of CysC and eGFRcys that might affect the clinicians’ decision for adopting CysC for the renal dose adjustment, were included in the full non-parsimonious model for CysC-guided dosing in propensity score estimation. To create the propensity score, multiple imputation with Markov Chain Monte Carlo methods was used to fill out incomplete baseline variables with the assumption that data were missing at random [22]. We employed the standardized difference (SD) to check for differences in the baseline characteristics. For categorical variables, we used a multivariate Mahalanobis distance method to generalize the standardized difference metric. It has been suggested that a SD of < 10% probably denotes a negligible imbalance. Differences in the survival rate and cumulative incidences of the cefepime-associated morbidity between the CysC-guided and SCr-guided groups were constructed using a Kaplan–Meier curve. Multivariable regression was performed using backward elimination with *p*-value < 0.2 on univariate analysis. All reported *p*-values are two sided, and *p*-values < 0.05 were considered statistically significant. Data manipulation and statistical analyses were conducted using SAS^®^ software version 9.4 (SAS Institute, Inc, Cary, NC, USA).

## 3. Results

### 3.1. Study Patients

**Figure 1:**
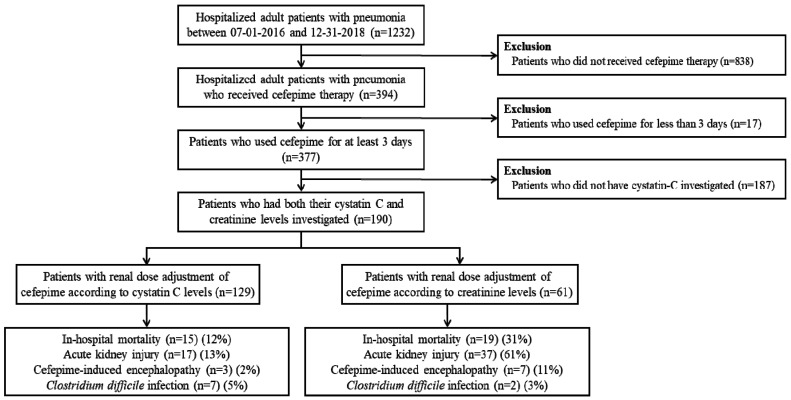
Flow diagram of the study.

In total, 1232 adults were diagnosed with pneumonia from July 2016 to December 2018 in Chung-Ang University Hospital (Figure 1). Eight hundred and thirty-eight individuals who did not use cefepime were excluded, as were 17 subjects who received cefepime for less than 3 days. Additionally, 187 people who did not have their CysC checked were excluded. Finally, 190 patients who simultaneously had their CysC and SCr checked were included. Finally, 190 patients who simultaneously had their CysC and SCr checked were included. Among these patients, 129 were administered cefepime with renal dose adjustment according to their CysC level, and the remaining 61 received renal dose adjustment of the drug according to their SCr level.

### 3.2. Baseline Clinical Characteristics of Patients and Outcomes

The baseline clinical characteristics and outcomes of the CysC-guided and SCr-guided group are shown in Table 1. There were no significant differences in age and gender between the two groups. However, compared with the SCr-guided group, the CysC-guided group had a higher BMI. Malignancy was more common in the SCr-guided group than in the CysC-guided group, but no significant differences were found in other underlying diseases. Regarding the laboratory findings, white blood cell, platelet, total bilirubin, and C-reactive protein levels were comparable in the both groups. However, the CysC-guided group had a higher blood urea nitrogen level than the SCr-guided group. Whereas eGFRcr did not significantly differ between the two groups, eGFRcys was higher in the CysC-guided group than in the SCr-guided group (*p* < 0.001). Shock was not significantly different between the two groups, but mechanical ventilation was more frequently applied in the SCr-guided group than in the CysC-guided group. Durations of cefepime, glycopeptides, and fluoroquinolone use were similar in the two groups. After propensity score-weighting, the standardized difference for each adjusted variable was < 10% except for BMI, classification of pneumonia, malignancy, platelet, blood urea nitrogen, CysC, and CysC-based eGFR (Table 1). Standardized differences of the covariates before and after IPTW are graphically presented in Supplement Figure S1.

### 3.3. Efficacy and Safety of Cystatin C-Guided Renal Dose Adjustment of Cefepime

**Figure 2:**
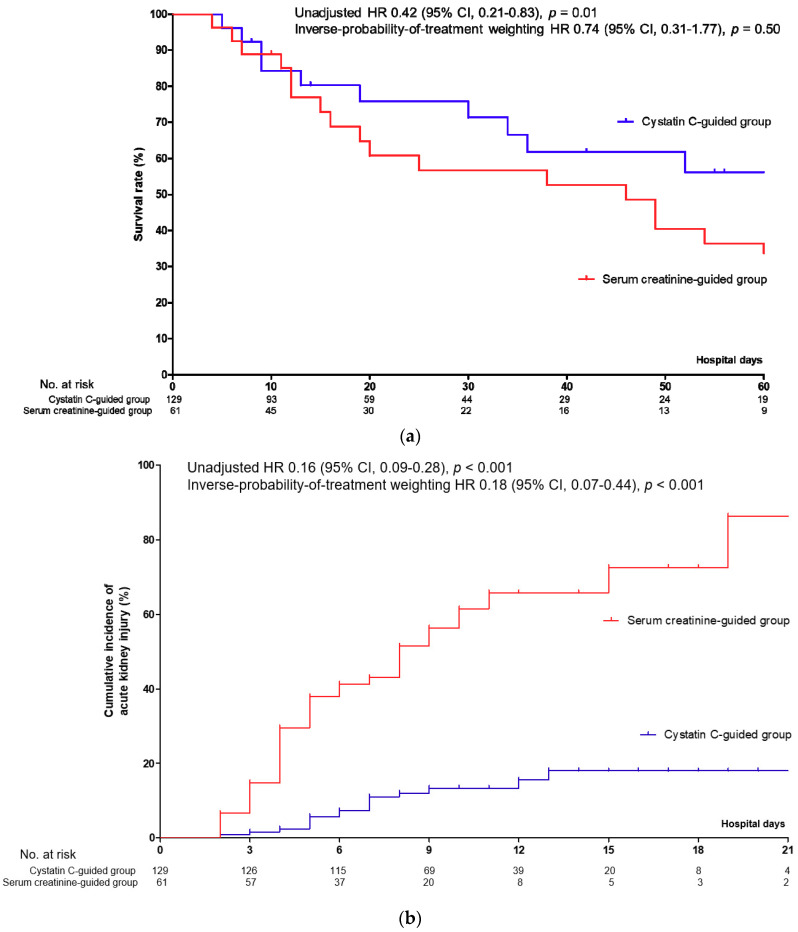

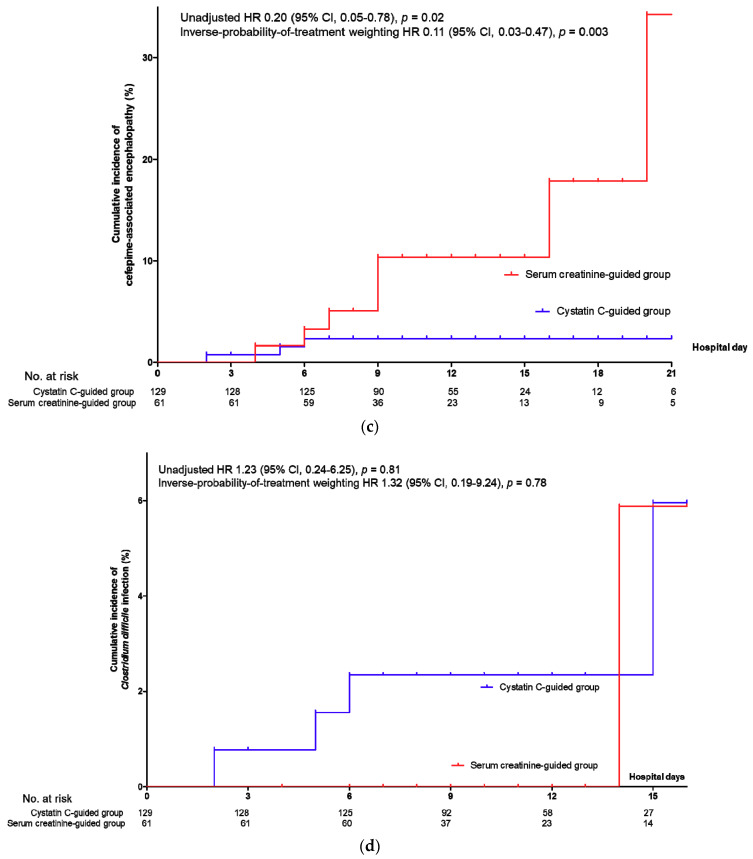
Difference in mortality and cefepime-associated morbidities among hospitalized patients with pneumonia. (**a**) Survival rates among patients in the cystatin C-guided and serum creatinine-guided groups. Cumulative incidence of acute kidney injury (**b**), cefepime-associated encephalopathy (**c**), and *Clostridium difficile* infection (**d**) among patients in the cystatin C-guided and serum creatinine-guided groups. Abbreviations: CI, confidential intervals; HR, hazard ratio.

The efficacy and safety of CysC-guided renal dose adjustment of cefepime are shown in Table 2. In unadjusted analysis, 14 day (hazard ratio (HR) 0.42; 95% confidence intervals (CI), 0.21–0.83) (*p* = 0.01), 60 day (HR 0.30; 95% CI, 0.11–0.82) (*p* = 0.02), and in-hospital (HR 0.33; 95% CI, 0.15–0.72) (*p* = 0.01) mortality were lower in the CysC-guided group than the SCr-guided group. AKI (HR 0.16; 95% CI, 0.09–0.28) (*p* < 0.001), severe AKI (HR 0.06; 95% CI, 0.02–0.17) (*p* < 0.001), and CIE (HR 0.20; 95% CI, 0.05–0.78) (*p* = 0.02) developed less frequently in the CysC-guided group compared with the SCr-guided group. However, the incidence of *Clostridium difficile* infection was not significantly different between the groups (HR 1.23; 95% CI, 0.24–8.25) (*p* = 0.81). Differences in the survival rate and the cumulative incidences of AKI, CIE, and *Clostridium difficile* infection between the CysC-guided and SCr-guided groups are shown in Figure 2a–d.

In the multivariable analysis, CysC-guided renal dose adjustment of cefepime was associated with a decreased risk of 60-day (HR 0.36; 95% CI, 0.15–0.87) (*p* = 0.02), and in-hospital (HR 0.40; 95% CI, 0.18–0.85) (*p* = 0.02) mortality. CysC-guided dosing of cefepime was also associated with a decreased risk of AKI (HR 0.15; 95% CI, 0.08–0.27) (*p* < 0.001), severe AKI (HR 0.07; 95% CI, 0.02–0.19) (*p* < 0.001), and CIE (HR 0.24; 95% CI, 0.06–1.01) (*p* = 0.05). However, the risk of *Clostridium difficile* infection did not significantly differ between the two groups (HR 0.66; 95% CI, 0.08–5.47) (*p* = 0.70).

In the IPTW analysis, the mortality at the various time points did not significantly differ between the two groups: HRs for 14 day, 30 day, 60 day, and in-hospital mortality were 0.75 (95% CI, 0.16–3.61, *p* = 0.72), 0.62 (95% CI, 0.15–2.56, *p* = 0.51), 0.56 (95% CI, 0.20–1.57, *p* = 0.27), and 0.68 (95% CI, 0.29–1.61, *p* = 0.38). respectively. CysC-guided renal dose adjustment of cefepime decreased the risk of AKI (HR 0.19; 95% CI, 0.08–0.48) (*p* < 0.001), severe AKI (HR 0.09; 95% CI, 0.02–0.38) (*p* = 0.001), and CIE (HR 0.14; 95% CI, 0.03–0.54) (*p* = 0.01). However, CysC-guided cefepime dosing did not reduce the incidence of *Clostridium difficile* infection (HR 1.32; 95% CI, 0.25–7.05) (*p* = 0.75). In the further analysis adjusted by IPTW and covariates with standardized difference of > 15% such as BMI, malignancy, blood urea nitrogen, and CysC, CysC-guided dosing decreased the risk of AKI (HR 0.18; 95% CI, 0.07–0.44) (*p* < 0.001), severe AKI (HR 0.08; 95% CI, 0.02–0.41) (*p* = 0.002), and CIE (HR 0.11; 95% CI, 0.03–0.47) (*p* = 0.003) without increasing in-hospital mortality (HR 0.74; 95% CI, 0.31–1.77) (*p* = 0.50).

## 4. Discussion

We have compared the mortality and the cefepime-associated morbidities between hospitalized patients with pneumonia who received CysC-guided and SCr-guided dosing of cefepime treatment. Compared with SCr-guided renal dose adjustment, CysC-guided renal dose adjustment decreased the risk of AKI and CIE by 82% and 89%, respectively, without increasing mortality in the present study. To the best of our knowledge, this is the first study evaluating the efficacy and safety of CysC-guided renal dose adjustment of cefepime.

Several pharmacokinetics studies have reported that CysC might more precisely predict vancomycin clearance and its serum concentration than SCr [23]. Furthermore, target trough drug concentration could be achieved more effectively by vancomycin dose adjustment according to CysC especially in critically ill patients [24,25]. In addition, eGFRcys might also be useful for estimating the pharmacokinetics of amikacin [26] and teicoplanin [27]. These might be partially explained by the fact that SCr could lead to overestimation of GFR in various conditions such as old age, malnutrition, low muscle mass, and hepatic dysfunction [14,15] and that CysC might more sensitively capture subtle fluctuations of GFR in critically ill patients than SCr [24]. However, there have been limited data on the clinical impacts of CysC-guided renal dose adjustment of cefepime in the context of the efficacy and safety. Therefore, our findings provide important evidence that cefepime could be used more safely by employing CysC-guided renal dose adjustment.

We found that AKI was common in hospitalized patients with pneumonia who were treated with cefepime. In total, 54 (28%) of the 190 patients included in our study developed AKI. The AKI has been studied mainly in comparison with AKI that was associated with piperacillin-tazobactam use wherein each drug was commonly used in combination with vancomycin. The incidence of AKI associated with cefepime use has been reported variably ranging from 11% to 29% [20,21,28,29]. The proposed mechanism of the cefepime-associated AKI was interstitial nephritis and direct cytotoxic damage with dose-dependent manner [7,8,9]. Although two meta-analyses have showed that piperacillin-tazobactam plus vancomycin led to a higher risk of AKI than cefepime plus vancomycin, the difference was not significant in critically ill patients [30,31]. This finding might reflect that AKI might be substantial depending on the morbidity of the patients and severity of the infection. Our findings suggest that a significant number of AKI cases might be preventable via CysC-guided renal dose adjustment of cefepime.

Impaired renal function and drug overdose have been well recognized as risk factors for CIE [10,12,32]. Cefepime might bind competitively to the γ-aminobutyric acid-A receptor [32]. Altered integrity of blood–brain barrier due to systemic inflammation and decreased renal clearance of cefepime could elevate the concentration of the drug in cerebrospinal fluid. However, CIE has been shown to develop despite renal adjusted dosing of the drug [32]. Given our finding that eGFRcys was lower than eGFRcr in the CysC-guided group in the present study, the patients in whom cefepime dose was adjusted according to SCr in the previous studies still might had been exposed to cefepime overdose. Especially, the patients whose SCr does not properly reflect renal function due to low muscle mass are easily exposed to adverse events caused by overdose of the drug as renal insufficiency is masked by overestimation of eGFR. Therefore, clinicians should keep in mind that patients might be administered an excessive dose of cefepime when cefepime was dosed according to eGFRcr. In these cases, it may be a safe option to check eGFR by measuring CysC together and drug dosing accordingly. However, CysC-guided renal dose adjustment of cefepime did not reduce the risk of *Clostridium difficile* infection in our study. This could be in partial because CysC-guided and SCr-guided groups both received glycopeptides and fluoroquinolones in combination with cefepime; these antibiotics could also attribute to the development of *Clostridium difficile* infection.

Our study has a few limitations. First, we could not check serum cefepime concentration in our patients because therapeutic drug monitoring of cefepime was not available in South Korea. Recently, Boschung-Pasquier et al. showed that trough cefepime level of ≧ 38.1 mg/L always led to CIE, although CIE was not observed among those with a cefepime level of < 7.7 mg/L [10]. Therefore, further pharmacokinetic studies evaluating the predictiveness of CysC for estimating the serum concentration of cefepime are needed. Second, a large number of patients were excluded especially in the process of including only those who checked both CysC and SCr. Further investigation is needed through a prospective cohort that includes CysC as a basic test to overcome the selection bias. Finally, given the lower BMI, higher proportion of malignancy, and more frequent mechanical ventilation in the SCr group, they might include more severe patients who are likely to prone to adverse outcomes. These imbalances between the two groups could affect the cefepime-associated morbidities. Therefore, a randomized controlled study is required to confirm the usefulness of CysC-guided renal dose adjustment of cefepime. However, as clinical evidence are insufficient, alternative study designs such as IPTW analysis are often needed to address important clinical questions.

## 5. Conclusions

In conclusion, compared with SCr-guided renal dose adjustment, CysC-guided renal dose adjustment of cefepime was associated with a decreased risk of cefepime-associated morbidities including AKI and CIE without increasing mortality in treatment of hospitalized patients with pneumonia. Our findings suggest that CysC could be a useful marker to use cefepime more safely that reduce the cefepime-associated morbidities without increasing mortality in treating pneumonia.

## Figures and Tables

**Table 1:** Clinical characteristics of patients with hospitalized pneumonia who received cefepime treatment with renal dose adjustment according to cystatin C and serum creatinine.

	Total Cohort Patients				Propensity-Score-Weighted Cohort Patients		
Variables	Cystatin C- Guided Group (*n* = 129)	Serum Creatinine-Guided Group (*n* = 61)	SD (%)	*p*-Value	Cystatin C- Guided Group (*n* = 129)	Serum Creatinine-Guided Group (*n* = 61)	SD (%)
**Age, mean years ± SD**	76 ± 13	78 ± 10	19.9	0.37	76 ± 13	77 ± 12	6.6
**Male sex**	81 (63)	39 (64)	2.4	0.88	81 (63)	38 (62)	1.1
**Body mass index, mean kg/m^2^ ± SD**	21.7 ± 4.0	20.3 ± 4.2	35.9	0.04	21.3 ± 3.9	20.5 ± 4.2	19.8
**Classification of pneumonia**							
Community-acquired pneumonia	18 (14)	4 (7)	24.6	0.14	16 (13)	5 (8)	14.4
Hospital-acquired pneumonia	99 (77)	49 (80)	24.6	0.14	100 (77)	50 (82)	14.4
Ventilator-associated pneumonia	12 (9)	8 (13)	12.1	0.42	13 (10)	6 (10)	0.3
**Underlying diseases**							
Neurologic disease	73 (57)	26 (43)	28.2	0.07	68 (53)	29 (48)	10.4
Diabetes	40 (31)	20 (33)	3.8	0.81	41 (32)	20 (33)	2.9
Cardiovascular disease	35 (27)	12 (20)	17.7	0.27	32 (25)	14 (22)	5.6
Chronic lung disease	31 (24)	16 (26)	5.1	0.74	31 (24)	15 (24)	1.2
Chronic kidney disease	28 (22)	17 (28)	14.3	0.35	32 (25)	15 (25)	1.1
Malignancy	26 (20)	24 (39)	42.9	0.01	35 (27)	21 (35)	16.9
Hyperthyroidism or hypothyroidism	2 (2)	2 (3)	11.3	0.60	2 (2)	1 (2)	2.2
**Laboratory findings**							
White blood cell, mean /mm^3^ ± SD	11,000 ± 5200	11,700 ± 6200	12.5	0.41	11,100 ± 5400	11,300 ± 5700	2.3
Leukocytosis (WBC > 10,000 /mm^3^)	72 (56)	35 (57)	3.2	0.84	73 (57)	35 (57)	0.3
Leukopenia (WBC < 4000 /mm^3^)	12 (9)	5 (8)	3.9	0.80	12 (10)	7 (11)	5.9
Platelet, × 10^3^ mean /mm^3^ ± SD	230 ± 115	207 ± 103	21.1	0.19	223 ± 114	208 ± 100	14.8
Thrombocytopenia (platelet < 150 × 10^3^ /mm^3^)	30 (23)	19 (31)	17.8	0.25	32 (24)	16 (26)	2.5
Blood urea nitrogen, mean mg/dL ± SD	23.4 ± 13.5	34.1 ± 25.4	52.8	<0.001	25.8 ± 16.4	28.9 ± 19.5	17.2
Serum creatinine, mean mg/dL ± SD	0.53 ± 0.37	0.61 ± 0.44	18.7	0.21	0.56 ± 0.39	0.57 ± 0.41	2.9
Creatinine-based eGFR, mean mL/min/1.73m^2^ ± SD	109 ± 38	100 ± 30	26.7	0.15	107 ± 38	103 ± 29	10.6
Cystatin C, mean mg/L ± SD	1.36 ± 0.79	1.86 ± 0.62	69.7	<0.001	1.44 ± 0.86	1.76 ± 0.6	42.5
Cystatin C-based eGFR, mean mL/min/1.73m^2^ ± SD	61 ± 28	36 ± 16	113.1	<0.001	59 ± 28	39 ± 17	84.0
Total bilirubin, mean mg/dL ± SD	0.7 ± 0.5	0.6 ± 0.5	12.3	0.47	0.7 ± 0.5	0.7 ± 0.6	3.0
C-reactive protein, mean mg/dL ± SD	14.1 ± 9.2	13.8 ± 9.6	3.4	0.83	13.6 ± 9.2	13.8 ± 9.1	1.7
**Shock**	18 (14)	13 (21)	19.4	0.20	19 (15)	9 (14)	2.1
**Mechanical ventilation**	23 (18)	21 (34)	38.5	0.01	27 (21)	14 (23)	4.1
**Duration of antibiotics treatment**							
Cefepime, mean days ± SD	9.1 ± 4.2	9.1 ± 4.9	1.5	0.71	9.0 ± 4.0	8.9 ± 4.6	1.7
Vancomycin or teicoplanin, mean days ± SD	1.5 ± 3.5	1.6 ± 4.0	1.6	0.99	1.5 ± 3.5	1.4 ± 3.6	2.2
Fluoroquinolone, mean days ± SD	3.5 ± 4.6	4.1 ± 5.5	11.9	0.62	3.6 ± 4.5	3.8 ± 5.0	3.5

Abbreviations: IQR = interquartile range; SD = Standardized difference. NOTE. Data are presented as the number (%) of patients unless otherwise indicated.

**Table 2:** Efficacy and safety of cystatin C-guided renal dose adjustment of cefepime in hospitalized patients with pneumonia.

	Cystatin C-Guided Group (*n* = 129)	Serum Creatinine-Guided Group (*n* = 61)	Unadjusted Analysis		Multivariable Analysis		IPTW Analysis		Adjusted by IPTW and Covariates ^a^	
			Hazard Ratio (95% CI)	*p*-Value	Hazard Ratio (95% CI)	*p*-Value	Hazard Ratio (95% CI)	*p*-Value	Hazard Ratio (95% CI)	*p*-Value
**Mortality**										
14 days mortality	5 (4)	6 (10)	0.40 (0.12–1.30)	0.13	0.55 (0.11–2.70)	0.46	0.60 (0.16–2.34)	0.47	1.03 (0.20–5.27)	0.98
30 days mortality	6 (5)	10 (16)	0.30 (0.11–0.82)	0.02	0.38 (0.10–1.37)	0.14	0.57 (0.18–1.79)	0.34	0.65 (0.16–2.69)	0.55
60 days mortality	10 (8)	16 (26)	0.33 (0.15–0.72)	0.01	0.36 (0.15–0.87)	0.02	0.46 (0.19–1.14)	0.09	0.60 (0.20–1.74)	0.34
In hospital mortality	15 (12)	19 (31)	0.42 (0.21–0.83)	0.01	0.40 (0.18–0.85)	0.02	0.57 (0.26–1.23)	0.15	0.74 (0.31–1.77)	0.50
**Cefepime-associated morbidities**										
Acute kidney injury	17 (13)	37 (61)	0.16 (0.09–0.28)	<0.001	0.15 (0.08–0.27)	<0.001	0.21 (0.11–0.40)	<0.001	0.18 (0.07–0.44)	<0.001
Severe ^b^ acute kidney injury	4 (3)	23 (38)	0.06 (0.02–0.17)	<0.001	0.07 (0.02–0.19)	<0.001	0.08 (0.02–0.28)	<0.001	0.08 (0.02–0.41)	0.002
Cefepime-induced encephalopathy	3 (2)	7 (11)	0.20 (0.05–0.78)	0.02	0.24 (0.06–1.01)	0.05	0.18 (0.04–0.77)	0.02	0.11 (0.03–0.47)	0.003
*Clostridium difficile* infection	7 (5)	2 (3)	1.23 (0.24–6.25)	0.81	0.66 (0.08–5.47)	0.70	1.09 (0.20–5.96)	0.92	1.32 (0.19–9.24)	0.78

Abbreviations: CI = confidential intervals; IPTW = inverse-probability-of-treatment weighting. ^a^ Adjustment was done through the use of the IPTW and covariates with standardized difference of >15% such as body mass index, malignancy, blood urea nitrogen, and cystatin C. ^b^ Severe acute kidney injury was defined as an increase in serum creatinine of 2.0–2.9 times from the baseline level (acute kidney injury stage II) and an increase in serum creatinine more than 3.0 times from the baseline level, ≥4.0 mg/dL or the initiation of renal replacement therapy (acute kidney injury stage III).

## Supplementary Materials

The following are available online at https://www.mdpi.com/2077-0383/9/9/2803/s1, Figure S1: Standardized differences of the covariates before and after IPTW.
