# Supplementary figures and images for: Efficacy and Safety of Cystatin C-Guided Renal Dose Adjustment of Cefepime Treatment in Hospitalized Patients with Pneumonia
Source: J Clin Med. 2020 Aug 30;9(9):2803. doi: 10.3390/jcm9092803 (PMC7564664; doi:10.3390/jcm9092803)

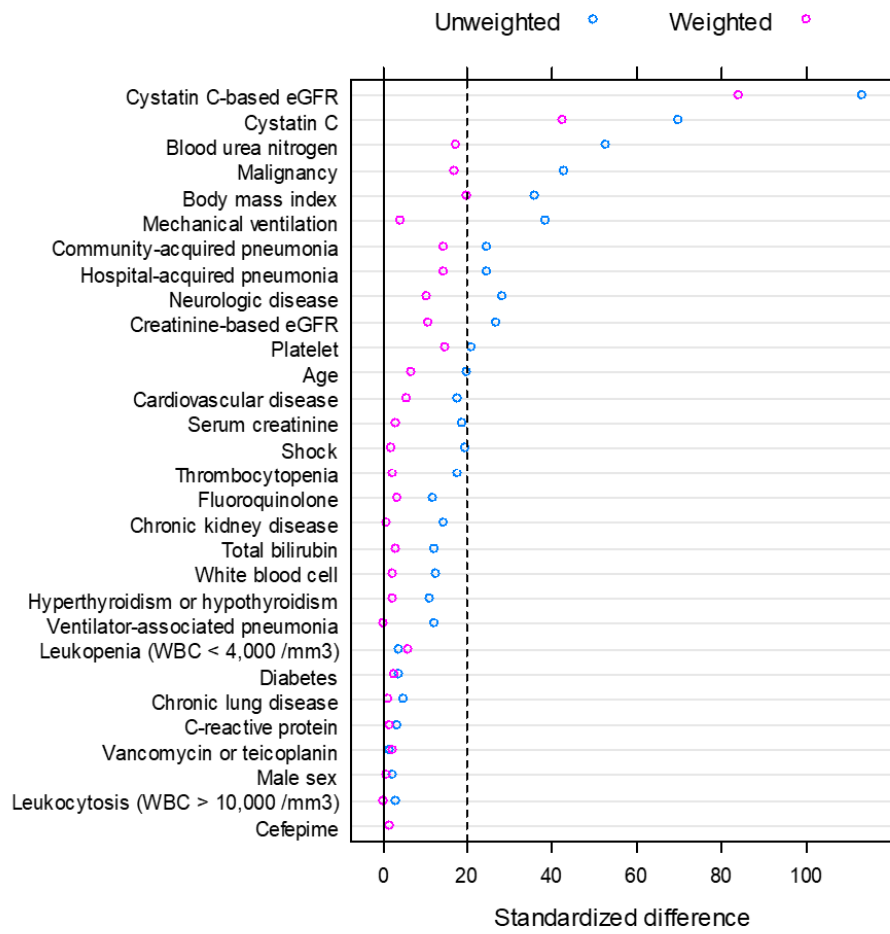

**Supplement Figure 1:** Standardized differences of the covariates before and after IPTW.

Supplement: Supplementary file 1 [file jcm-09-02803-s001.pdf]
